# Supplementary material for: Single Excited Dual Band Luminescent Hybrid Carbon Dots-Terbium Chelate Nanothermometer
Source: Nanomaterials (Basel). 2021 Nov 15;11(11):3080. doi: 10.3390/nano11113080 (PMC8618998; doi:10.3390/nano11113080)
Supplement: Supplementary file 1 [file nanomaterials-11-03080-s001.zip › nanomaterials-1452383-supplementary.pdf]

Supplementary Materials

Single Excited Dual Band Luminescent Hybrid Carbon Dots-Terbium Chelate Nanothermometer

Rustem R. Zairov [1,*], Alexey P. Dovzhenko [2], Kirill A. Sarkanich [2], Irek R. Nizameev [3], Andrey V. Luzhetskiy [4], Svetlana N. Sudakova [1], Sergey N. Podyachev [1], Vladimir A. Burilov [2], Ivan M. Vatsouro [5], Alberto Vomiero [6,7] and Asiya R. Mustafina [1]

[1] Arbuzov Institute of Organic and Physical Chemistry, FRC Kazan Scientific Center, Russian Academy of Sciences, Arbuzov Str., 8, 420088 Kazan, Russia; sudakova@iopc.ru (S.N.S.); spodyachev@gmail.com (S.N.P.); asiyamust@mail.ru (A.R.M.)

[2] Department of physical chemistry, Kazan (Volga Region) Federal University, Kremlyovskaya Str., 18, 420008 Kazan, Russia; aleksej_dovghenko@mail.ru (A.P.D.); sarkanich98@mail.ru (K.A.S.); ultrav@bk.ru (V.A.B.)

[3] Department of Nanotechnologies in electronics, Kazan National Research Technical University Named after A.N. Tupolev-KAI, 10, K. Marx Str., 420111 Kazan, Russia; irek.rash@gmail.com

[4] Federal State Autonomous Educational Institution of Higher Education "Gubkin Russian State University of Oil and Gas" (National Research University), Leninsky Prospect, 65, 119991 Moscow, Russia; luzhetskiy@yandex.ru

[5] Department of Chemistry, M. V. Lomonosov Moscow State University, Lenin's Hills 1, 119991 Moscow, Russia; vatsouro@petrol.chem.msu.ru

[6] Department of Molecular Sciences and Nanosystems, Ca' Foscari University Venezia, Via Torino 155, 30172 Venezia-Mestre, Italy; alberto.vomiero@ltu.se

[7] Division of Materials Science, Department of Engineering Sciences and Mathematics, Luleå University of Technology, SE-971 87 Luleå, Sweden

* Correspondence: rustem@iopc.ru; Tel.: +7-843-2734573; Fax: +7-843-2731872

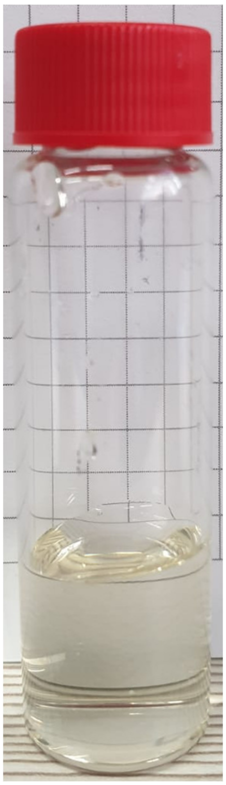

Figure S1. Image of CDs dispersion in water (C=10 μg mL^{-1}).

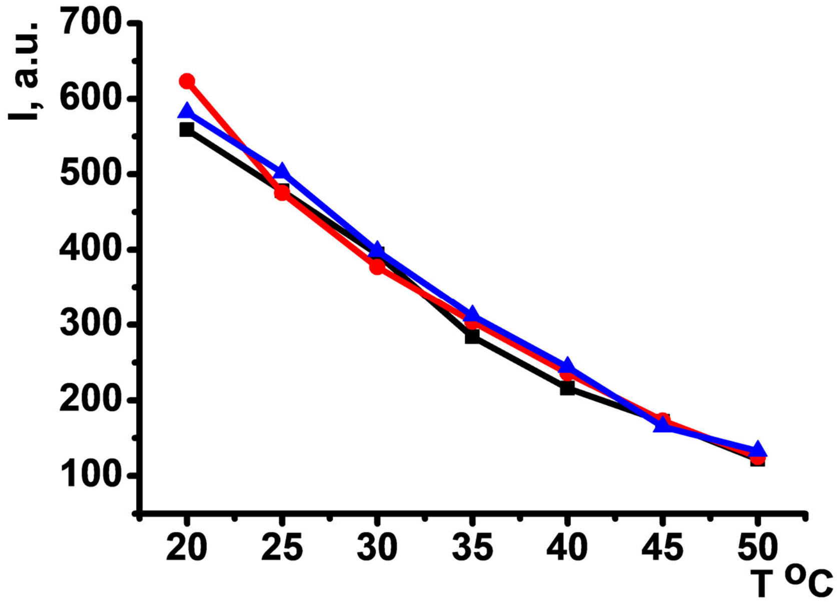

Figure S2. Luminescence intensity of $[TbL]^{+}$ (l_{em}=547 nm) versus temperature during heating-cooling-heating cycle in DMF (C_L =C_{Tb} =1·10^{-4} M).

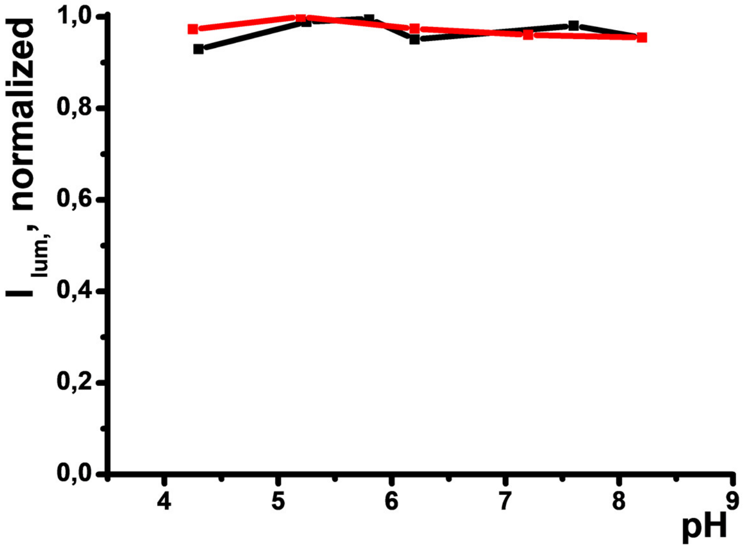

Figure S3. pH-dependency of CDs (lem=450 nm, C=0.01 g/L) in physiological range of pH=4.5–8.0.

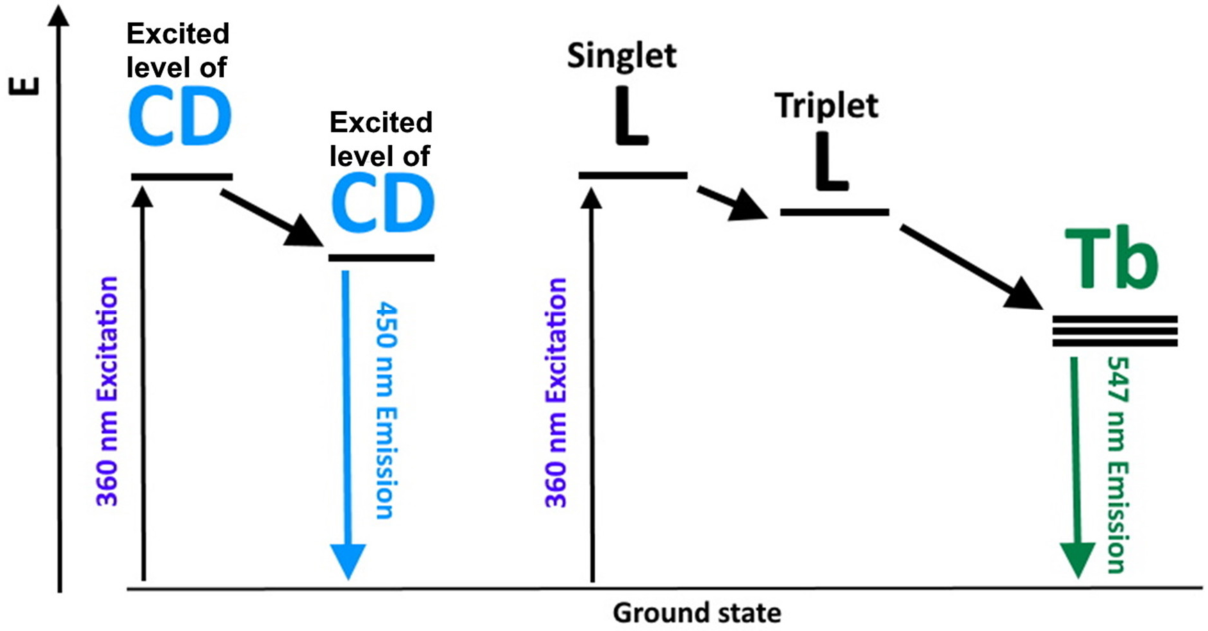

Figure S4. Tentative Jablonski diagram showing photophysical processes responsible for generation of dual band emission of PSS-{CDs-[TbL]} colloids.

Table S1. Luinescence decay exponential deconvolution data and lifetimes of $[TbL]^{+}$ at different CDs in DMF (C_{CD}=0.09-3.15 mg·L^{-1}; $C_{[TbL]}$=0.45 mM).

C_{CD}	y_0	A_1	◎ms
0.09	2.057	1495.2	0.896
0.18	2.076	1409.4	0.914
0.27	2.447	1449.8	0.920
0.36	2.258	1568.7	0.932
0.45	2.159	1560.3	0.964
1.2	2.302	1552.3	0.968
1.4	2.263	1507.7	0.974
1.6	2.416	1500.8	0.976
1.8	2.731	1566.7	0.989
0.9	2.999	1571.7	0.980
1.35	2.729	1474.6	1.016
1.8	3.202	1499.5	1.032
2.25	2.922	1499.9	1.048
3.15	3.102	1511.1	1.072

Table S2. Average lifetimes of Tb(III) excited state for PSS-{CDs-[TbL]} colloids obtained according to *Synthesis_1* and *Synthesis_2* in water.

	A1	t1	A2	t2	t_{avg}, ms	Adj. R-Square
Synthesis_1	1.282	0.068	0.321	0.305	0.193	0.99704
Synthesis_2	1.161	0.087	0.356	0.309	0.203	0.99985

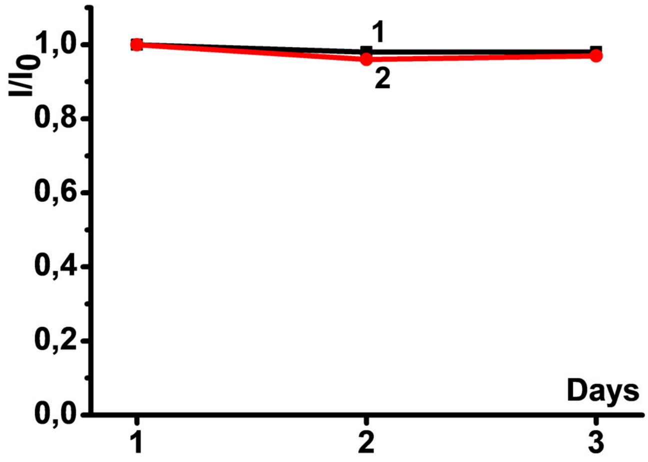

Figure S5. The luminescence intensity of PSS-{CDs-[TbL]} water colloids for each of 3 days of storage (I) related to initial luminescence intensity (I_0) at emission wavelengths 450 nm (1) and 547 nm (2).

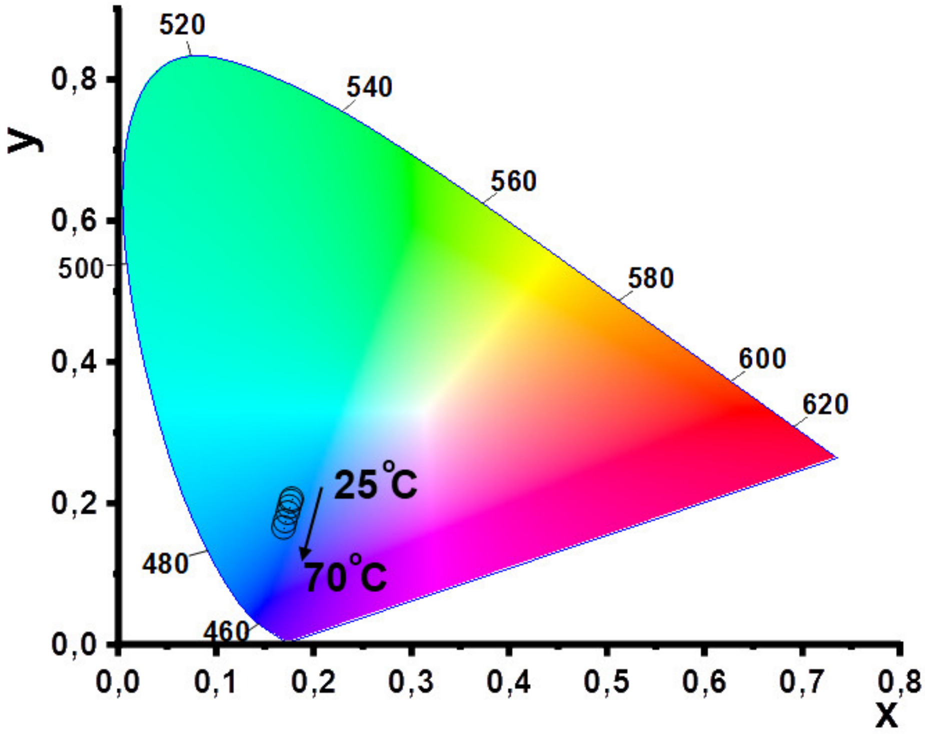

Figure S6. Chromaticity diagram for PSS-{CDs-[TbL]} (*Synthesis_1*) (l$_{ex}$ =360 nm) as a function of temperature over the 25–70°C range.
